# Anterior gradient protein 2 expression in high grade head and neck squamous cell carcinoma correlated with cancer stem cell and epithelial mesenchymal transition

**DOI:** 10.18632/oncotarget.3556

**Published:** 2015-03-12

**Authors:** Si-Rui Ma, Wei-Ming Wang, Cong-Fa Huang, Wen-Feng Zhang, Zhi-Jun Sun

**Affiliations:** ^1^ The State Key Laboratory Breeding Base of Basic Science of Stomatology & Key Laboratory of Oral Biomedicine Ministry of Education, Wuhan University, Wuhan, China; ^2^ Department of Oral Maxillofacial-Head Neck Oncology, School and Hospital of Stomatology, Wuhan University, Wuhan, China

**Keywords:** AGR2, HNSCC, cancer stemloid cell, epithelial mesenchymal transition

## Abstract

Anterior gradient protein 2 (AGR2) is a novel biomarker with potential oncogenic role. We sought to investigate the diagnostic and prognostic role of AGR2 on head and neck squamous cell carcinoma (HNSCC) with an emphasis on its correlation of cancer stemloid cells (CSC) and epithelial mesenchymal transition (EMT). We found that AGR2 protein levels were higher in HNSCC than in normal oral mucosa. High levels of AGR2 were associated with the T category, pathological grade and lymph node metastasis of HNSCC. Expression of AGR2 increased in recurring HNSCC after radiotherapy and in post cisplatin-based chemotherapeutic tissues. In HNSCC cell lines, knock-down of AGR2 induced apoptosis, reduced sphere formation, and down-regulated Survivin, Cyclin D1, Bcl2, Bcl2l1, Slug, Snail, Nanog and Oct4. In addition, over-expressed AGR2 in transgenic mice with spontaneous HNSCC was associated with lost function of *Tgfbr1* and/or lost function of *Pten*. *In vitro* knockdown TGFBR1 in HNSCC cell lines increased AGR2 expression. These results suggest that AGR2 is involved in EMT and self-renewal of CSC and may present a potential therapeutic target (oncotarget) for HNSCC.

## INTRODUCTION

Cancer stem cells (CSC) or cancer initiating cell are thought to be responsible for tumor metastasis, chemoresistance, radioresistance and recurrence [1]. The CSC theory suggests that a part of cancer cells with self-renewal and multi-potential differentiation abilities are responsible for the maintenance and progression of tumors [2, 3]. Existing researches showed that a small group of proteins can be regarded as CSC markers. ALDH1, a detoxifying enzyme that oxidizes intracellular aldehydes, was reportedly a promising cancer stem cell marker in numerous cancers comprising head and neck squamous cell carcinoma [4]. Over expressions of Sox2 and Oct 4 in numerous malignancies were also reportedly related to the pluripotency, proliferation, and self-renewal properties of embryonic stem cells (ESCs) and germ cells [5]. Epithelial-mesenchymal-transition (EMT) is an evolutionarily developmental process [6]. Epithelial cells gain mesenchymal characteristics and lose epithelial characteristics, thereby becoming more invasive and with stronger motility [7]. Accumulated studies demonstrated that EMT is a common event in malignancies, and contributes to the invasion process and cancer stem cell development. Previous studies showed that metastasis cancer cells, which probably underwent EMT, could exhibit CSC phenotype [2]. Evidence suggests that the Snail family plays crucial roles in inducing EMT in cancers [8]. Moreover, aberrant expression of Snail2 (Slug) can induce radio resistance in cancer cells [8]. Snail and Slug effectively mediate cell survival and are involved in the acquisition of a self-renewal CSC-like trait (e.g., NANOG, HDAC1, DHAC3, TCF4, KLF4, and GPC3) [8].

Head and neck squamous cell carcinoma (HNSCC), with more than 450,000 newly diagnosed cases every year, is the 6^th^ most common cancer around the world [9]. Despite intense research efforts, the morbidity and mortality of HNSCC has remained nearly unchanged for the past several decades [10]. As the oral cavity is the most common site (almost 90%) of HNSCC [9], curative systemic therapies for oral squamous cell carcinoma (OSCC) are still lacking. Recurrence, cervical lymph node metastasis, and radiotherapy and chemotherapy resistance may be the most important factors affecting the poor prognosis of HNSCC [11]. Thus, discovering the mechanism underlying disease initiation and progression is important. New biomarkers, especially HNSCC stem cell molecular therapeutic targets, are urgently needed.

Anterior gradient protein 2 (AGR2), the human homolog of the *Xenopus laevis*-secreted protein XAG-2, is strongly expressed in tissues that contain mucus secreting cells [12]. Emerging evidences showed that abnormal expression of AGR2 might be involved in tumorigenesis and progression of human cancers, such as prostate, breast, ovarian, esophagus, gastro-intestinal tract, and lung cancers, as reviewed by Brychtova et al. in Ref [13]. Increased AGR2 expression contributes to metastasis because of its capacity to enhance migration of breast cancer and oral squamous cell carcinoma *in vitro* and *in vivo* [14-16]. Recent reports showed that aberrant AGR2 expression helps breast cancer cells survive under serum-depleted conditions and/or hypoxic culture conditions, promotes angiogenesis, and increases cell invasion [17]. AGR2 can regulate breast cancer cells growth and survival by modulating Survivin, C-myc, and Cyclin D1 [18]. AGR2 is reportedly a P53 suppressor and subsequently promotes cancer metastasis; AGR2 is correlated with negative prognosis of cancer patients [19-22]. These findings highlight the importance of AGR2 in cancer initiation, progression, migration, and metastasis. However, the underlying mechanism and biological implication of AGR2, especially in cancer stem cell and epithelial mesenchymal transition, remain unclear.

In this study, we aimed to characterize the expression of AGR2 in human HNSCC tissue arrays and to further determine the correlation and role of AGR2 in cancer stem cell and EMT by *in vitro* functional assay and *in vivo* observation using transgenic mice HNSCC models.

## RESULTS

### Expression of AGR2 is positively related to high grade human HNSCC

**Figure 1:**
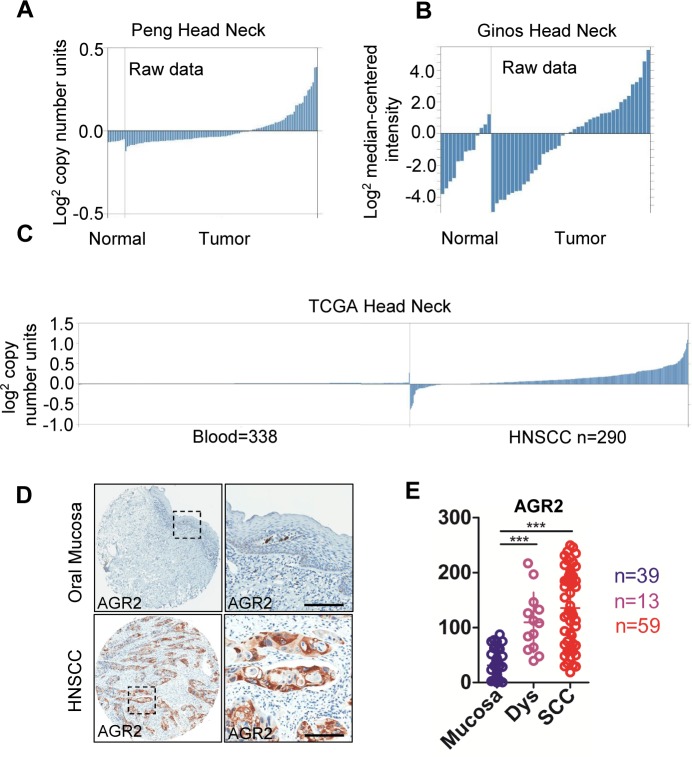
AGR2 expression in human head neck cancer (A) *AGR2* DNA copy number from Peng's dataset and (B) mRNA expression from Ginos's dataset (as log2 median –centered ratio) for head neck cancer versus normal counterpart shown as raw data. (C) *AGR2* DNA copy number of TCGA head neck cancer as shown as raw data. (D) Representative immunohistochemistry staining of AGR2 in oral mucosa as well as in HNSCC tissue with quantification in (E, One way ANOVA*, P* <0.001). Dys, dysplasia; SCC, squamous cell carcinoma; ***, *P* <0.001.

**Figure 2:**
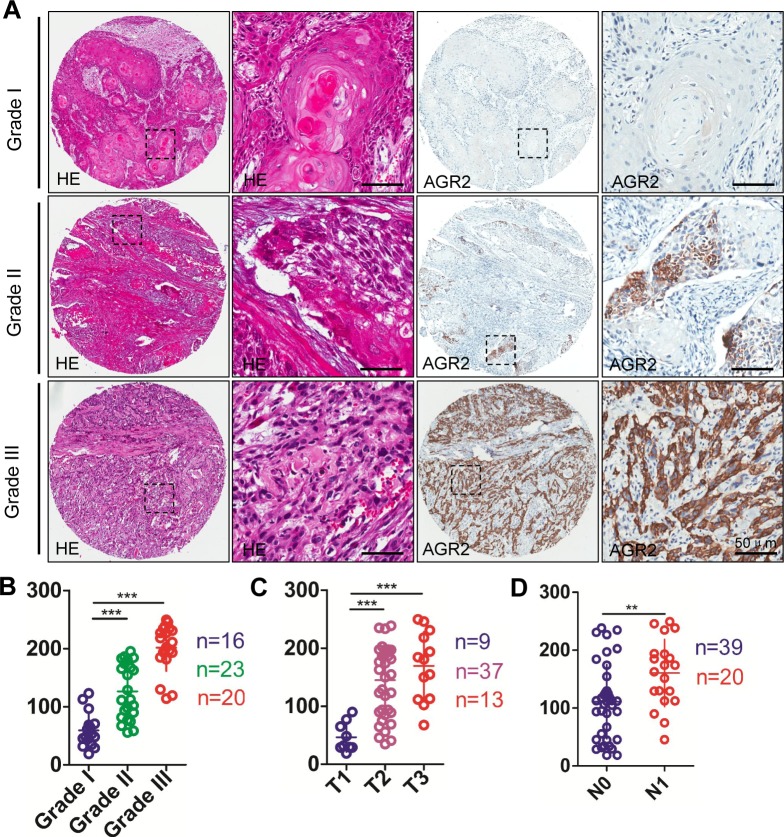
Human HNSCC tissue array analysis revealed that AGR2 correlated with high grade HNSCCs (A) Representative hematoxylin-eosin staining (HE, left) and immunohistochemical (IHC) staining (right) of AGR2 in human oral cancer tissues with different grades (I-III) (Scale bars =50um); (B) Quantitative analysis of histoscore of AGR2 expression in normal oral mucosa, oral epithelial dysplasia and human HNSCC. AGR2 levels in HNSCC and/or oral epithelial dysplasia was significant higher when compared with normal oral mucosa (One way ANOVA*,P* < 0.001); (C) The expression of AGR2 was correlated with T category of human oral cancer(One way ANOVA, *P* < 0.001); (D) The expression of AGR2 was correlated with lymph node metastasis of human HNSCC (*t* test, *P* < 0.01). **, *P* <0.01; ***, *P* <0.001. Scale bars=50um

To determine whether *AGR2* expression was associated with HNSCC in humans, we interrogated the Tissue Cancer Genome Atlas dataset [23] and Oncomine database [24]. In a meta-analysis of gene expression profiling, increased AGR2 DNA copy number of mRNA expression was significantly increased in many cancers (e.g., esophagus, thyroid, ovarian, pancreatic, breast, prostate, lung, and HNSCC) compared with the normal counterpart (*P* < 0.001, Supplementary Fig. 1), thereby suggesting that *AGR2* may act as an oncogene in human cancer cells. In the Peng et al. dataset [25], which independently performed DNA copy number analysis on oral squamous cell carcinoma, 38 out of 112 OSCCs showed amplification of *AGR2* copy number (Fig. 1A). Through analyze the raw data of Ginos et al. dataset [26] using Oncomine, we found the significantly enhanced mRNA expression of *AGR2* in 21 out of 54 HNSCCs compared with normal oral mucosa (Fig. 1B). TCGA data sheet indicated an increase in the DNA copy number of HNSCC (n = 290) compared with the normal counterpart (blood, n = 338, Fig. 1C). Furthermore, to assess the protein expression of AGR2 in human HNSCC tissues, we performed immunohistochemistry in human HNSCC tissue microarray (Fig. 1D). AGR2 exhibited high expression in the cytoplasm and membrane of the cancer cells (Fig. 1D). This analysis showed significantly increased immunoreactivity of AGR2 in HNSCC (n = 59) compared with dysplasia (n = 13, *P* < 0.001) and normal oral mucosa (n = 39, *P* < 0.001, Fig. 1D with quantification in Fig. 1E). High-grade (Grades II and III) HNSCC presented intense AGR2 immunoreactivity compared with low-grade (Grade I, n=20) HNSCC (*P* < 0.001, Fig. 2A with quantities in Fig. 2B). The expression of AGR2 was also associated with tumor size and with increased AGR2 expression in T2 (n = 37) and T3 category (n = 13) compared with the T1 category (n = 9, Fig. 2C). We also noted a remarkable increase in AGR2 immunoreactivity in the original tumor of the pathological lymph node-positive patient (pN1, n = 20, *P* < 0.01, Fig. 2D) compared with the pathological lymph node-negative patient (pN0, n = 39). The results above indicated that AGR2 protein expression correlated with high-grade HNSCC. To further explore the prognosis value of AGR2 in HNSCC, Kaplan–Meier method was used. As shown in Supplementary Fig. S2, high AGR2 expression may indicate a rather poor prognosis of HNSCC patient, whereas log-Rank analysis indicated that cumulative rate of the patients with high AGR2 (*P =* 0.1161, n = 29) expression did not reach statistical significance.

### Radiotherapy and chemotherapy dramatically induced AGR2 expression in HNSCC tissue

**Figure 3:**
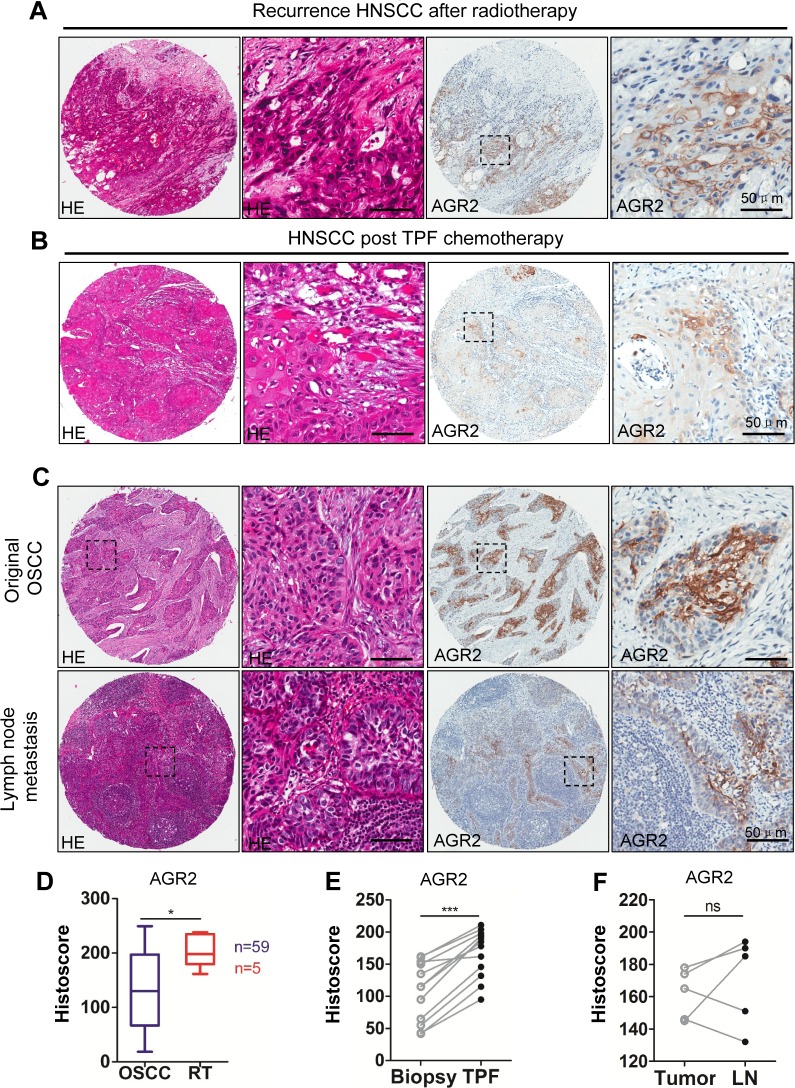
Increased expression of AGR2 in recurrence HNSCC after radiotherapy, cisplatin based chemotherapy and lymph node metastasis (A) Representative HE staining (left) and immunohistochemical staining of recurrence HNSCC (right) after radiotherapy; (B) Representative HE staining (left) and immunohistochemical staining (right) of recurrence HNSCC after combined cisplatin, docetaxel, and fluorouracil (TPF) chemotherapy; (C) Representative HE staining (left) and immunohistochemical (right) staining of original HNSCC and lymph node metastasis;(D) Quantitative of immunohistochemistry satinning using digital scanner and histoscore of AGR2 expression after radiotherapy was significantly higher than original HNSCC (*t* test, *P* < 0.05). (E) The expression level of AGR2 in recurrence after TPF chemotherapy was significantly higher than original HNSCC (paired *t* test, *P* < 0.001); (F) There was no significant difference between the expression level of AGR2 in original HNSCC and lymph node metastasis(*t* test, *P>0.05*). Scale bars=50um

To further investigate the protein expression of AGR2 in post-radiotherapy recurrence HNSCC, chemotherapeutic response and lymph node metastasis of HNSCC, five recurrence cases after radiotherapy and 12 paired HNSCC cases, including presurgical biopsy and postsurgical specimen of two rounds of TPF (cisplatin, docetaxel, and fluorouracil) chemotherapy were selected for immunohistochemistry analysis. Immunohistochemistry was performed in original tumor and in paired lymph node metastasis (n = 5). Representative hematoxylin-eosin staining and immunohistochemistry photos are shown in Figs. 3A and 3B. Pathologically, recurrent HNSCC after radiotherapy usually presents as high-grade and poorly differentiated SCC with spindle-shaped epithelial cells and intense hematoxylin-stained nuclear area (Fig. 3A left). Immunohistochemistry showed a significant increase of AGR2 expression in recurrent HNSCC after radiotherapy (*P* < 0.05, Fig. 3A with quantification in Fig. 3D). Pathologically, HNSCC specimen with inductive TPF chemotherapy showed local regression of the epithelial island and increased local immunoreactivity to AGR2 compared with paired biopsy (n = 12, *P* < 0.001, Fig. 3B with quantification in Fig. 3E). No significant difference was found between the original tumor and lymph node metastasis (*P >* 0.05, Fig. 3C and quantification in Fig. 3F), which may be due to the significant increase of AGR2 in the original tumor of the node-positive patient (Fig. 3C). The results above revealed that radiotherapy and chemotherapy may induce AGR2 expression.

### Protein expression of AGR2 was remarkably correlated with Survivin, Cyclin D1, ALDH1, Sox2, Oct4, and Slug in HNSCC tissue

**Figure 4:**
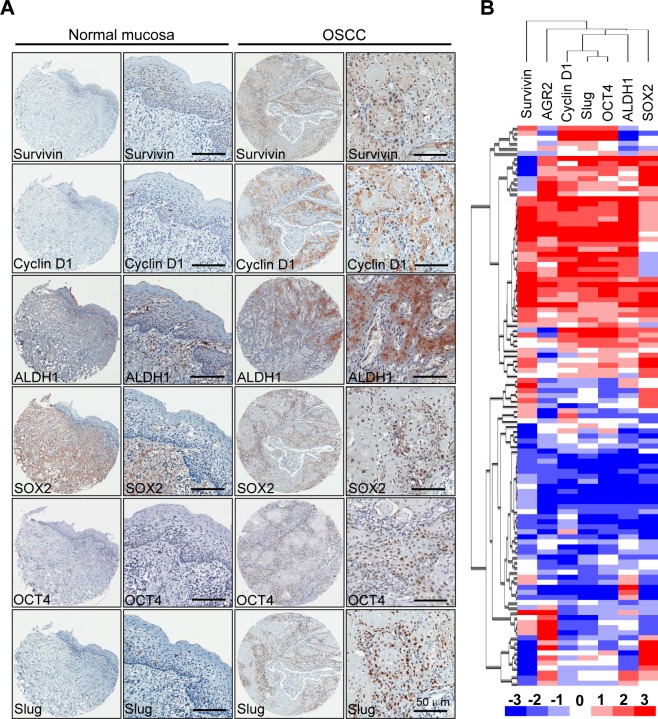
Correlation of Survivin, Cyclin D1, ALDH1, Oct4, Sox2 and Slug in human HNSCC tissue array (A) Representative immunohistochemical staining (IHC) of AGR2, Survivin, Cyclin D1, ALDH1, Oct4 and Slug in human oral cancer tissue (right) compared with normal oral mucosa (left) (Scale bars =50um); (B) Hierarchical clustering present the protein expression correlation of AGR2, Survivin, Cyclin D1, ALDH1, Sox2, Oct4 and Slug in human HNSCC tissue array.

Extensive studies have demonstrated that cancer stem cells are abundant in chemotherapy and radiotherapy, which prompted our study on the correlation of AGR2 with self-renewal and pro-survival factors. Immunohistochemistry with specific antibody was used to stain the human HNSCC tissue arrays. As shown in Fig. 4A, an increase in Survivin and Cyclin D1 in the cytoplasmic and nuclear areas of HNSCCs compared with normal mucosa was observed. Expression of ALDH1 was mainly in the cytoplasm, whereas the over expression of Sox2, Oct4, and Slug was mainly found in the nuclear. Afterward, Spearman rank correlation coefficient test and linear tendency test were conducted. We found that the protein expression of AGR2 in HNSCC was significantly correlated with Survivin (*P <* 0.01, r = 0.2466), Cyclin D1 (*P <* 0.001, r = 0.4425), ALDH1 (*P* < 0.001, r = 0.5508), Sox2 (*P* < 0.001, r = 0.4573), Oct4 (*P* < 0.001, r = 0.5606), and Slug (*P* < 0.001, r = 0.5214) (Supplementary Figs. S3, Supplementary Table 2). In addition, by using hierarchical clustering analysis, we found that the expressions of pro-survival marker Survivin and CSC marker were close to the expression of AGR2 (Fig. 4B). The observation above suggested that AGR2 might play a key role in pro-survival, EMT, and self-renewal, which is a characteristic of cancer stem cells.

### Knocked down AGR2 effectively decreased colony formation, sphere formation and cancer stem cell marker in human HNSCC cell line

**Figure 5:**
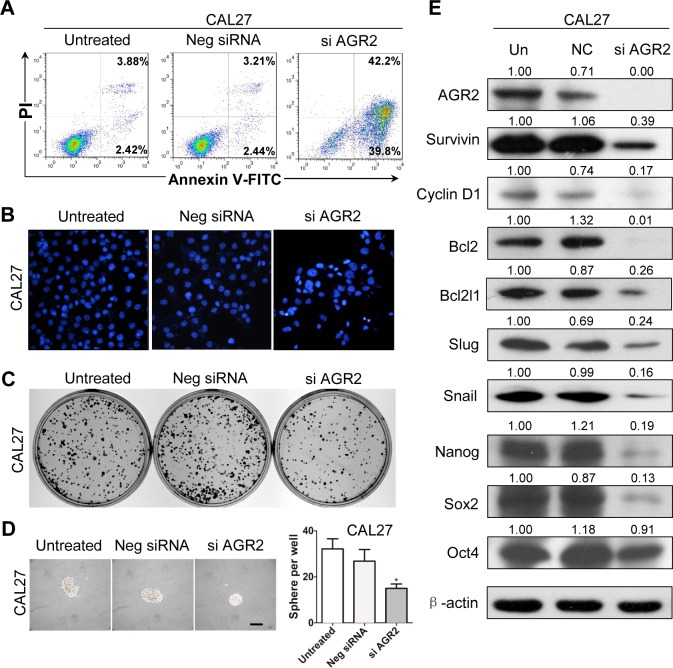
Knock down of AGR2 induces cell apoptosis and reduces colony formation in CAL27 cell line (A) Annexin V-FITC/PI dual labeling assay showed AGR2 siRNA enhance apoptosis in CAL27 cell lines by using flow cytometry; (B) The morphologic changes of CAL27 cell lines transfected with AGR2 siRNA was observed by fluorescence microscopy with DAPI staining; (C) Knock down of AGR2 in CAL27 cell line reduced anchor dependent colony formation; (D) Knock down of AGR2 in CAL27 cell line reduced sphere formation; Scale bar=100μm;(E) Western blot analysis revealed that the protein level of Survivin, Cyclin D1, Bcl2, Bcl2l1, Slug, Snail, Nanog, Sox2 and OCT4 were reduced in different degrees after AGR2 konck down for 48h. Quantification is performed using Image J by pixel analysis of band by normalized of β-actin as a loading control. Neg siRNA, negative siRNA,si AGR2, AGR2 siRNA.

To direct identify the function of AGR2, we used siRNA to knockdown AGR2 in human HNSCC CAL27 and FaDu cell lines. By using Annexin V/PI double staining analysis, cell apoptosis was counted by flow cytometry. Annexin V^+^/PI^−^ apoptotic cell population increased in AGR2 siRNA-treated CAL27 cell line compared with the untreated and negative control siRNA counterpart (Fig. 5A). Hoechst staining of siAGR2 increased the nuclear fragment as shown in Fig. 5B. The colony formation ability and sphere formation assay is widely used as a surrogate for self-renewal and colony formation property of CSCs. Therefore, we performed the sphere-forming assay to investigate the potential role of AGR2 knock down in the self-renewal capacity of CAL27 cells. Indeed, AGR2 siRNA reduced the colony formation (Fig. 5C) when compared with the untreated group and negative group. AGR2 siRNA treated CAL27 cells was found to form less and smaller spheres than the counterpart (Fig. 5D) indicating its possible inhibition of self-renewal of HNSCC cells *in vitro*. The apoptotic assay and sphere formation assay are quiet repeatable in FaDu cell line (Supplementary Fig. S4 A-C)

To verify whether AGR2 knockdown downregulated the protein expressions of CSC and EMT markers, we performed a Western blot analysis in the whole-cell lysate collected from CAL27 cell and FaDu cell line with or without AGR2 siRNA treatment. We found that AGR2 siRNA consistently reduced pro-survival markers (Survivin, Bcl2, Bcl2l1, and Cyclin D1), stem cell markers (Nanog, Sox2 and OCT4), and EMT markers (Slug and Snail, Fig. 5E and Supplementary Fig. 4D) compared with the negative control counterpart. Immunofluorescence assay further showed that AGR2 could regulate Survivin and Cyclin D1 in CAL27 cell line (Supplementary Figs. S5 A to C). The results support the hypothesis that AGR2 knockdown can effectively inhibit the self-renewal capacity of human HNSCC cells *in vitro*.

### Overexpression of AGR2 is associated with *Tgfbr1* deletion and high grade transformation *in vitro* and *in vivo*

**Figure 6:**
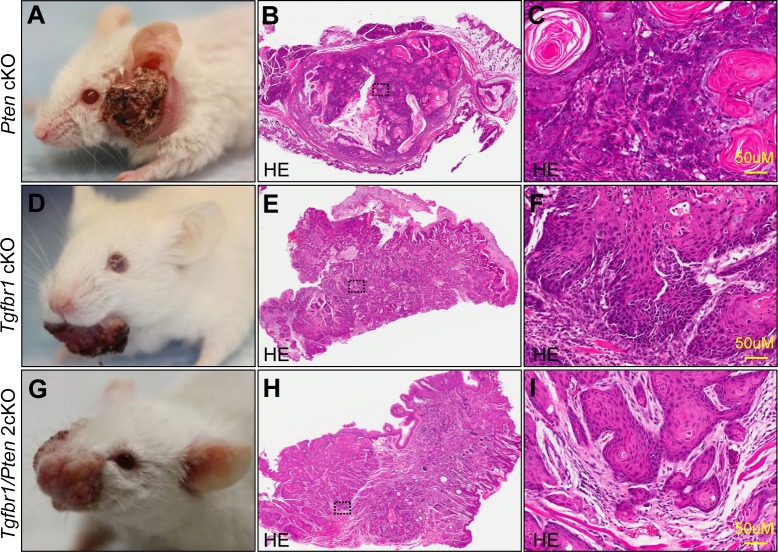
*Tgfbr1* deletion related with high grade mice HNSCC Pathologically, *Pten* cKO mice HNSCC (A) are well differentiated squamous cell carcinoma (B and high magnificence in C). Conditional knock out Tgfbr1 with single dose application of DMBA induced HNSCC (D) pathological present as poor differentiated spindle type epithelial malignancy, with extensive inflammation, abundant stromal cells and less keratin pear (E and high magnificence in F). Combined deletion of *Tgfbr1/Pten* in mice epithelial and oral mucosa will lead to a fast HNSCC formation (G), with pathological present as poor differentiated SCC (H and high magnificence in I). Scale bar=50μm.

**Figure 7:**
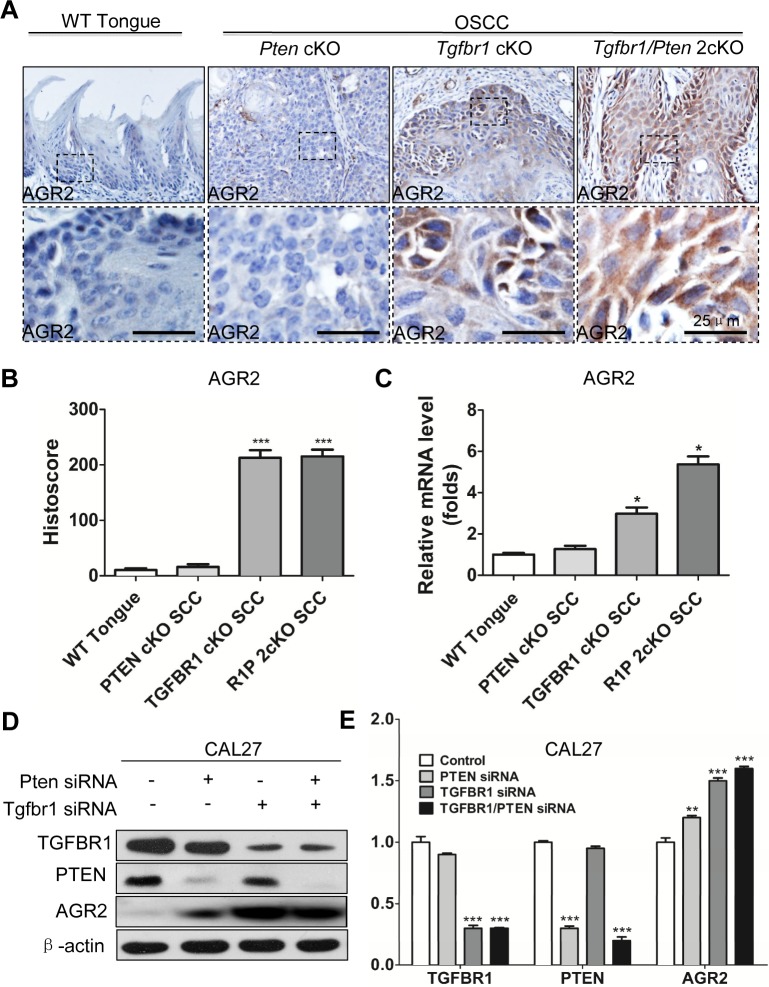
Increased expression of AGR2 is associated with *Tgfbr1* deletion (A) Immunohistochemistry of AGR2 in tongue of the wild type (WT) mice, the tumor of *Pten* conditional knock out mice, *Tgfbr1* conditional knock out mice and *Pten/Tgfbr1* conditional knock out mice (Scale bar+25μm); (B) Quantitative of histoscore of AGR2 in wild type mice, *Pten* conditional knock out mice, *Tgfbr1* conditional knock out mice and *Pten/Tgfbr1* conditional knock out mice. Expression of AGR2 in *Tgfbr1* conditional knock out mice and *Pten/Tgfbr1* conditional knock out mice was significantly higher than the wild type mice (*P* < 0.001); (C) Quantitative Real-time PCR revealed the mRNA level of AGR2 in *Tgfbr1* conditional knock out mice and *Pten/Tgfbr1* conditional knock out mice was significantly increased when compared with the wild type mice; (D) Western blot analysis of AGR2 48h after knocking down PTEN, TGFBR1, and combined TGFBR1/PTEN by using siRNA; (E) Quantitative analysis showed the protein level of AGR2 in TGFBR1 siRNA group and TGFBR1/PTEN combined siRNA group were significantly higher than the control group (*P* < 0.001). Mean±SEM, ***, *P*<0.001.

Recent studies revealed that TGFβ signaling plays a pivotal role in EMT and in cancer stem cell self-renewal [27]. Our previous work showed that mice with tissue specific deletion of tumor suppressor gene *Pten* in epithalia had spontaneous HNSCC (Fig. 6A). Pathologically, *Pten* conditional kock out (*Pten* cKO) mice tumor present as well differentiation keratinizing squamous cell carcinoma (8/8) with frequently observed extracellular keratin, similar to human Grade I SCC (Fig. 6B and high magnification in Fig. 6C). *Tgfbr1*conditional knock out (*Tgfbr1*cKO) mice Fig. 6D) tumor histologically present as aggressive strand or island growth pattern (Fig.6E). The transitional epithelial cell present spindle-type with extensive inflammation, abundant stromal cells, intratumor necrosis and low amount of keratin pearl formation (High magnification in Fig. 6F), which similar to human Grades II and III squamous cell carcinoma. Combined deletion of *Tgfbr1/Pten* in mice epithelia would lead to rapid tumor formation (Fig. 6G) with pathological infiltrating growth pattern (Fig. 6H) and poorly differentiated SCC (High magnification in Fig. 6I). To determine whether over-expression of AGR2 was related to high-grade squamous cell carcinoma in mice, we performed AGR2 immunohistochemistry and found that AGR2 was located mostly in the membrane and cytoplasm of the cancer cell of *Tgfbr1* cKO mice and *Tgfbr1/Pten* 2cKO mice (n = 5, respectively). However, AGR2 staining was negative in wild type mice mucosa and focal weakly positive in *Pten* cKO mice HNSCC (Fig. 7A with quantification histoscore in Fig. 7B). The mRNA levels of AGR2 in *Tgfbr1* cKO mice and *Tgfbr1/Pten* mice were significantly higher as compared with wild type mice mucosa (*P* < 0.001 respectively, Fig. 7C). We further knockdown TGFBR1 and/or PTEN in human HNSCC CAL27 and FaDu cell lines. After transfection with PTEN and/or TGFBR1 siRNA for 48 h, the protein was extracted and analyzed by Western blot. The protein level of AGR2 in PTEN, TGFBR1 as well as in combined PTEN/TGFBR1 knock down was significantly higher than scramble siRNA in CAL27 cell line (Fig. 7D and quantification in Fig. 7E) and quiet repeatable in FaDu cell line Supplementary Fig. S4E and 4F). The discovery above suggested that increased AGR2 protein level may be associated with lost function of TGFBR1.

## DISCUSSION

The existence of CSCs has attracted a high amount of attention recently because they are major contributing factors to cancer recurrence and metastasis, which severely increase HNSCC morbidity and motility [2]. The multidrug and radiotherapy resistance phenotype of CSCs is responsible for radio-chemotherapy failure [28]. Recently, AGR2 expression is known to be regulated at multiple levels. Using the Oncomine database, we observed that AGR2 may serve as a promising oncogene in human cancer. HNSCC showed an increased AGR2 DNA copy number ratio as well as AGR2 mRNA level. Although mRNA and DNA copy numbers of AGR2 in HNSCC were not as significant as in other cancers. The protein expression of AGR2 in human HNSCC was consistent with the DNA copy number and the mRNA level, as indicated in this study as well as in other studies [14, 16]. The present study is the first report that described abnormal AGR2 expression in HNSCC with radiotherapy and chemotherapy.

For the first time, we demonstrated the increase of AGR2 protein in HNSCC in correlation with aggressive phenotype HNSCC, as indicated by high protein expression, high pathological grade, large tumor burden, positive lymph node status, and recurrence [13]. Moreover, remarkable associations were found between AGR2 and ALDH1, Sox2, Oct4, and Slug in human HNSCC tissue. The high expression of AGR2 in HNSCC in post-radiotherapy and post-chemotherapy tissues was in agreement with the phenotype of CSCs, thereby indicating that AGR2 may be associated with CSCs of HNSCC. This correlation was further validated by direct *in vitro* blockage of AGR2 using siRNA, which indicated that AGR2 inhibition decreased the function and the self-renewal markers of HNSCC CSCs. This finding is in agreement with several reports, which stated that in other cancers (breast, ovarian, and prostate cancer), AGR2 was a marker that was used to determine negative prognosis [20-22]. Nevertheless, in our present study, AGR2 was not significantly correlated with the prognosis of HNSCC patients, even if patients with high expression of AGR2 seemed to have longer survival time than those with low expression of AGR2. Larger scale of cases and longer follow up time are needed in future studies.

Interestingly, several reports indicated the close relationship of EMT with cancer stem cell line phenotype. Pathophysiological conditions, including tissue injury or tumorigenesis, may trigger a stem-like phenotype in differentiated cells during EMT induction [29]. This phenomenon may mirror the developmentally regulated EMT pathways, including Wnt, Notch, and Hedgehog, which also show CSC self-renewal and maintenance [30, 31]. A recent report indicated that AGR2 enhanced *in vitro* cell invasion and metastasis in HNSCC [14], Thus, we hypothesized that AGR2 might enhance cancer invasion by increasing EMT. Similar reports indicated that AGR2 could promote cellular proliferation and metastasis of HNSCC [16]. In our study, we verified the close relationship of AGR2 protein expression with surrogate EMT marker Slug in human HNSCC tissue. Additionally, knockdown of AGR2 *in vitro* significantly decreased the protein levels of two EMT-related transcription factors, namely, Snail and Slug. Using transgenic mice, we also observed that the increased expression of AGR2 was correlated with epithelial *Tgfbr1*-deleted mice HNSCC, which pathologically presents as high-grade SCC with the EMT phenotype. The tumors of *Tgfbr1* cKO and *Tgfbr1*/*Pten* 2cKO mice expressed high levels of AGR2 and presented a more aggressive phenotype. This finding was in agreement with the report that AGR2 was regulated by TGFβ1 and SMAD4 [32]. Our previous study suggested that the loss of *Tgfbr1* in epithelial cells would lead to the accumulation of TGFβ1 in stroma [33]. In the present study, an *in vitro* study revealed that AGR2 was regulated by loss of *Tgfbr1* in CAL27 cell lines.

Results of previous studies showed that AGR2 played an important role in cell proliferation, cell cycle invasion, and poor response to chemotherapy [34]. A recent report [35] suggested that AGR2 may promote cancer cell invasion by increasing the expressions of MUC1 and lysosomal enzymes cathespin B and cathepsin D; thus, AGR2 overexpression plays an important role in invasion, grading, and prognosis of HNSCC [36-38]. Serum MUC1 and MUC1 immunoreactivities were significantly correlated with grading and prognosis of HNSCC [39]. AGR2 overexpression in primary breast cancer is correlated with poor response to tamoxifen treatment, which is Akt dependent [40]. AGR2 may also suppress p53 by up-regulating MDM2 [34], which is an important molecular event in human HNSCC. In our study, we found that the overexpression of AGR2 was correlated with lymph node-positive status, which was also reported in gastric cancer [41]. Less known protein–protein interactions and upstream and downstream molecular regulation of AGR2 occur even in other species (e.g., fly or yeast, Supplementary Fig. S6). Several studies indicated that AGR2 may regulate c-Myc and Amphiregulin, as well as EGFR [42]. In the present study, we showed for the first time that *in vitro* knockdown AGR2 may be correlated with self-renewal markers, such as Sox2, ALDH1, and Oct4. The so called “cancer stem cell” in this study is actually proliferating cancer stem cell (cancer stemloid) [1, 43]. Our data indicated that AGR2 may regulate Survivin, an important member of the inhibitor-of-apoptosis family, which has been revealed to interfere with cell apoptosis. This observation agreed with the results of similar studies on breast cancer [18], thereby suggesting that inhibition of AGR2 reduced the protein level of Survivin and Cyclin D1.

In summary, we identified the clinical significance of AGR2 in human HNSCC tissues, and further showed that AGR2 promoted HNSCC progression and radio-chemotherapy by regulating CSC and EMT signaling pathways. The upstream regulation of TGFBR1 on The above data suggested AGR2 was also clarified. AGR2 is a potential therapeutic oncotarget of HNSCC.

## MATERIALs AND METHODS

### Cell culture, siRNA knock down assay and Hoechst 33258 staining

Human head neck cancer cell line CAL27 cells were bought from the American Type Culture Collection (Manassas, VA) in 2012 and maintained in DMEM with 10% fetal bovine serum at 5% CO­2 and 37°C in a humidified incubator. The CAL27 cell line was characterized and authenticated by STR method with 100% of match. TGFBR1 siRNA, PTEN siRNA, and combined TGFBR1/PTEN siRNA were transfected into appropriate cells using HiPerfect transfection reagent (Qiagen, Germantown, MD) with a final concentration of 5nM as previous described [44, 45]. AGR2 siRNA (Qiagen), were transfected in CAL27 cell in final concentration of 5nM. All-star negative controls (Qiagen) were used as negative controls to confirm there is no interference with other miRNAs. MAPK1 siRNA and Cell Death siRNA (Qiagen) were used as positive controls [44, 45]. For inhibition efficiency and target mRNA transcription studies, RNA was extracted 24 h after transfection. For protein extraction, cells were harvested 48 h after transfection. The morphologic changes of CAL27 treated with indicated siRNA were captured using fluorescence microscopy with Hoechst 33258 staining.

### Annexin V/PI staining

After being transfected AGR2 siRNA (Qiagen) in final concentration of 5nM, CAL27 cells were detached by trypsin-EDTA. Annexin V/PI (BD Pharmingen) staining was carried out following manufacture's instruction. The cells were then counted by flow cytometer as previous described [46].

### Colony formation assay and sphere formation assay

Anchorage-dependent *in vitro* colony formation assay were performed as previously described [45]. Briefly, CAL27 cells were seeded at a density of 600 cells per well in flat-bottomed six-well culture plates. After colonies were visible, the colonies were fixed by methanol and stained by crystal violet (Sigma–Aldrich, St. Louis, MO, USA). The percentage of cells that formed into a clone was calculated. CAL27 cells were treated with siAGR2 with counterpart control as described above. Sphere cell culture was preformed according to published protocol with minor modifications [47]. Briefly, single-cell suspensions were plated in six-well ultralow attachment plates (Corning Inc.) at a density of 1,000 cells/mL. Cells were maintained in serum-free DMEM/F12 containing negative siRNA and AGR2 siRNA with blank control. The number and size of tumor spheres formed were evaluated by light microscopy after 7 days.

### Cell immunofluorescence

In order to immunofluorescent staining, CAL27 cells were seeded on coverglass slide chambers (Millipore). After designed treatment, the cells were fixed by 4% paraformaldehyde at room temperature for 15min, and then treated with 0.3% triton X-100. After blocked with 2.5%BSA for 1h, the cells were incubated with primary antibody overnight at 4°C. Then cells were incubated with secondary antibodies with 4′, 6′-diamidino-2-phenylidole (DAPI) (Jackson ImmunoResearch Laboratories, Inc, West Grove, PA) for 1 hour in the dark at room temperature. The primary antibodies included the following: AGR2 antibody (Cell Signaling Technology, 1:200), Suvivin antibody (Cell Signaling Technology, 1:200), Cyclin D1 antibody (Epitomics, 1:200). The slides were observed by a fluorescent microscope. Representative cells were selected and photographed.

### Quantitative real-time PCR

miRNeasy Mini kit (Qiagen, Hilden, Germany) was used to extract the total RNA from Pten cKO, mice, *Tgfbr1* cKO mice, *Pten/Tgfbr1* 2cKO mice. RNA quality control was performed as previously described [44]. Mouse *Agr2* primer was purchased from Qiagen. The mRNAs were subjected to quantitative 2-step real-time PCR using invitrogen transcript III kit and SYBR green PCR kit (Bio-Rad). qRT-PCR was performed in triplicate on the Chrom 4 real-time PCR System (Bio-Rad, Hercules, CA) using a GAPDH probe as an internal control, according to the protocol suggested by the manufacturer.

### Western blot

CAL cell lines were treated with the indicated concentrations AGR2 siRNA, TGFBR1 siRNA, PTEN siRNA and TGFBR1/PTEN combined siRNA in DMEM for 48h. Then the cells were lysed, and the total protein was separated by using 12% SDS-polyacrylamide gel electrophoresis and then transferred onto polyvinylidene fluoride membranes (Millipore Corporation, Bil-lerica, MA). Then blots were developed by Enhanced chemiluminescence detection kit (West Pico, Thermo). GAPDH was detected on the same membrane and used as a loading control.

### Knock out mice HNSCC models

All experiments were conducted in accordance with guidelines of Institutional Animal Care and Use Committee of Wuhan University. Time inducible tissue specific *Tgfbr1/Pten* 2cKO mice (*K14-Cre*^ERtam^; *Tgfbr1*^flox/flox^; *Pten*^flox/flox^), *Tgfbr1* cKO mice (*K14-Cre*^ERtam^; *Tgfbr1*^flox/flox^), *Pten* cKO mice (K14-Cre^ERtam^; *Pten*^flox/flox^) were maintained and genotyped according to published protocols [44, 45]. All the mice were FVBN/CD1/129/C57 mixed background.

### Patients, tumor samples and human HNSCC tissue array

The School and Hospital of Stomatology of Wuhan University Medical Ethics Committee approved this study, and informed consent was obtained from the patients before they underwent surgery. 59 patients tissues with histologically confirmed HNSCC in the Hospital of Stomatology of Wuhan University between January 2008 and August 2010 were recruited. Their clinical and histological data are provided in Supplementary Table 1. The clinical stages of their HNSCC were classified according to the guidelines of the International Union Against Cancer (UICC 2002), and histological grading was determined according to the scheme of the World Health Organisation. Custom made tissue arrays of formalin-fixed tissues from HNSCC mentioned above were constructed with 1.5mm core from each patient (T12-412) [44] and T12-412-2) mentioned above. These tissue microarray slides included 59 confirmed cases of HNSCC, 39normal oral mucosa and 13 oral epithelial dysplasia, 5 paired lymph node metastasis, 5 recurrences after radiotherapy and 12 TPF chemotherapy. The 12 HNSCC patients receive 2 round combined cisplatin, docetaxel, and fluorouracil therapy with the same protocol of Zhang's clinical trial [48]. Patient samples with both biopsy as well as surgical specimen after 2 rounds were involved in these custom-made tissue microarrays.

### Immunohistochemical staining

All paraffin-embedded specimens were cut into 4-μm sections and dried at 60 °C for 2 h, deparaffinised and dehydrated. The sections were then boiled in 0.01 M citric acid buffer solution (pH 6.0) for 1.5 min at high pressure. After being incubated with 3% hydrogen superoxide for 20 minutes to quench endogenous peroxidase activity and 10% normal goat serum to block non-specific binding, the sections were incubated overnight at 4 °C with polyclonal rabbit anti-human AGR2 (Cell Signaling Technology, 1:200), Survivin (Cell Signaling Technology, 1:200), Cyclin D1 (Epitomics, 1:200), ALDH1 (Proteintech gruop, 1:100), (Epitomics, 1:200), OCT4 (Proteintech gruop, 1:100), Slug (Cell Signaling Technology, 1:200). Then, the sections were incubated with a secondary biotinylated immunoglobulin G antibody solution and an avidin-biotin-peroxidase reagent. After being washed with phosphate buffer saline, the sections were incubated with 3,3′-diaminobenzidine tetrachloride and then lightly counterstained with Mayer's haematoxylin.

### Scoring system, hierarchical clustering and data visualization

Whole slices were scanned using an Aperio ScanScope CS scanner (Vista, CA, USA) with background substrate for each slice, and quantified using Aperio Quantification software (Version 9.1) for membrane, nuclear, or pixel quantification [49]. An area of interest was selected either in the epithelial or the cancerous area for scanning and quantification. Histoscore of membrane and nuclear staining was calculated as a percentage of different positive cells using the formula (3+)×3+(2+)×2+(1+)×1. Histoscore of pixel quantification was calculated as total intensity/total cell number [44]. The threshold for scanning of different positive cells was set according to the standard controls provided by Aperio [33, 44]. The expression scores were converted into scaled values centered on zero in Microsoft excel. Then the hierarchical analysis was achieved by the Cluster 3.0 with average linkage based on Person's correlation coefficient [50]. Java TreeView 1.0.5 was used to visualize the results [51]. Finally, we arranged the clustered data and tissue samples on the horizontal axis and vertical axis respectively. Biomarkers with a close relationship are located next to each other.

### Statistical analysis

Data analyses were performed using Graph Pad Prism version 5.0 for Windows (Graph Pad Software Inc, La Jolla, CA). One-way ANOVA followed by the post-Tukey or Bonferroni multiple comparison tests were used to analyze the differences in immunostaining and protein levels among each group. Paired *t* test was used to analyze immunohistochemical staining of AGR2 in TPF chemotherapy specimen and difference between original HNSCC and metastatic lymph nodes. Two-tailed Pearson's statistics was used for correlated expression of AGR2, Survivin, Cyclin D1, ALDH1, Sox2, Oct4 and Slug after confirmation of the sample with Gaussian distribution. Survival curves were plotted using the method of Kaplan-Meier and the significance of observed differences was accessed with log-rank test. Mean values ± SEM with a difference of *P* < 0.05 were considered statistically significant.

## SUPPLEMENTARY MATERIAL, FIGURES AND TABLES


