# Supplementary material for: Anterior gradient protein 2 expression in high grade head and neck squamous cell carcinoma correlated with cancer stem cell and epithelial mesenchymal transition
Source: Oncotarget. 2015 Mar 12;6(11):8807–21. doi: 10.18632/oncotarget.3556 (PMC4496185; doi:10.18632/oncotarget.3556)
Supplement: Supplementary file 1 [file oncotarget-06-8807-s001.pdf]

Anterior gradient protein 2 expression in high grade head and neck squamous cell carcinoma correlated with cancer stem cell and epithelial mesenchymal transition

Supplementary Material

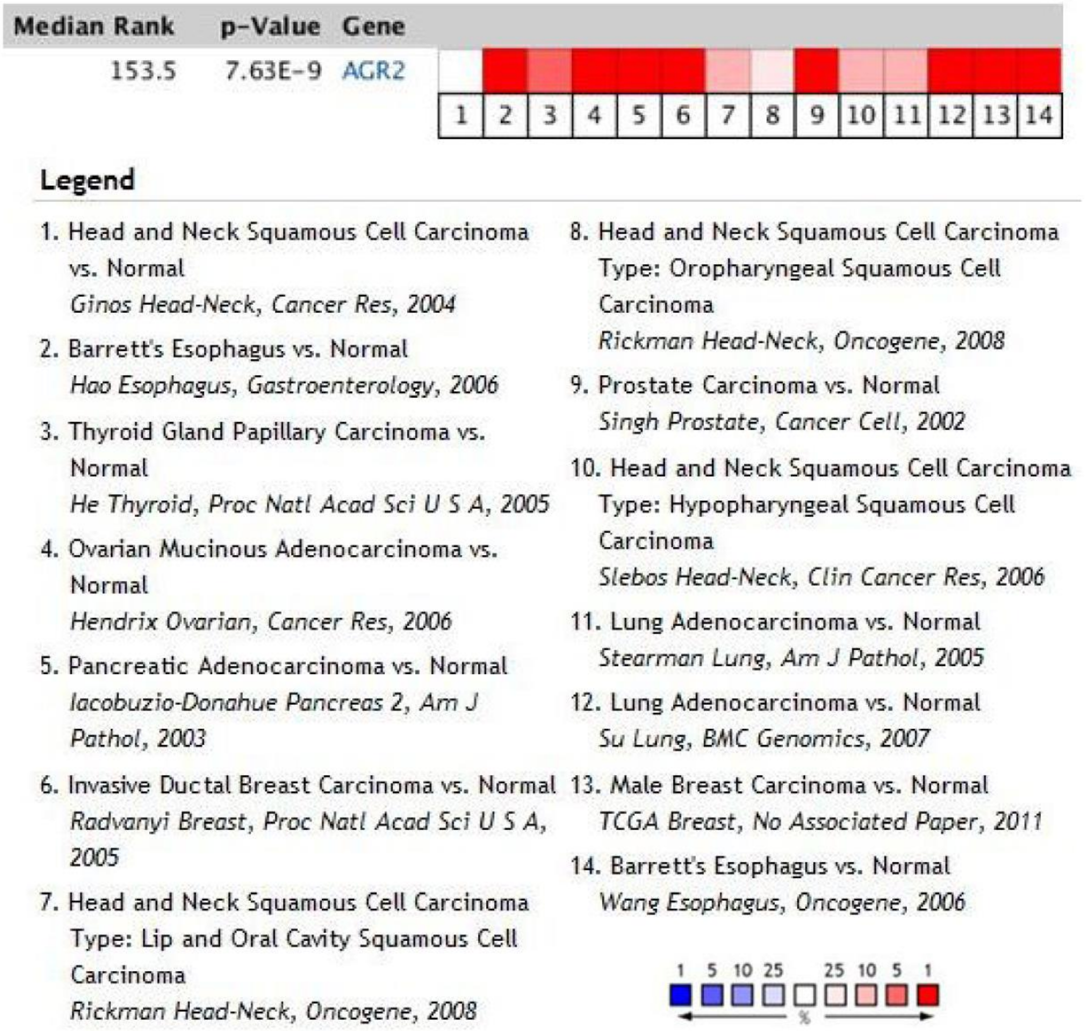

Supplementary Figure S1: AGR2 expression in human head neck cancer. Meta-analysis of recent gene expression profiling for *AGR2* where the colored squares indicated the median rank for AGR2 across each analysis in various human cancer.

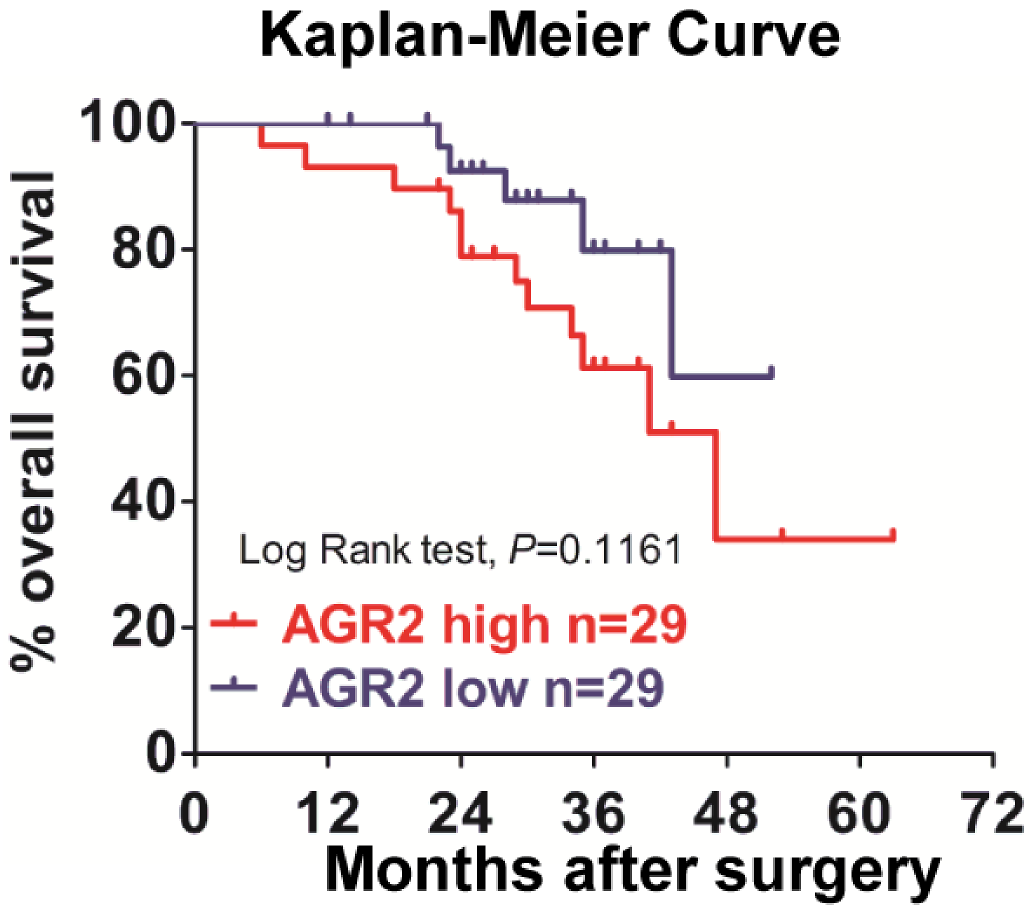

Supplementary Figure S2: AGR2 expression of correlated with poor survival of human HNSCC. Kaplan Meier curve showing head neck squamous cell carcinoma patient with low AGR2 expression (n=29) survival longer than AGR2 high expression patient, which log-Rank analysis reveal the difference was not significant (n=29, *P*>0.05).

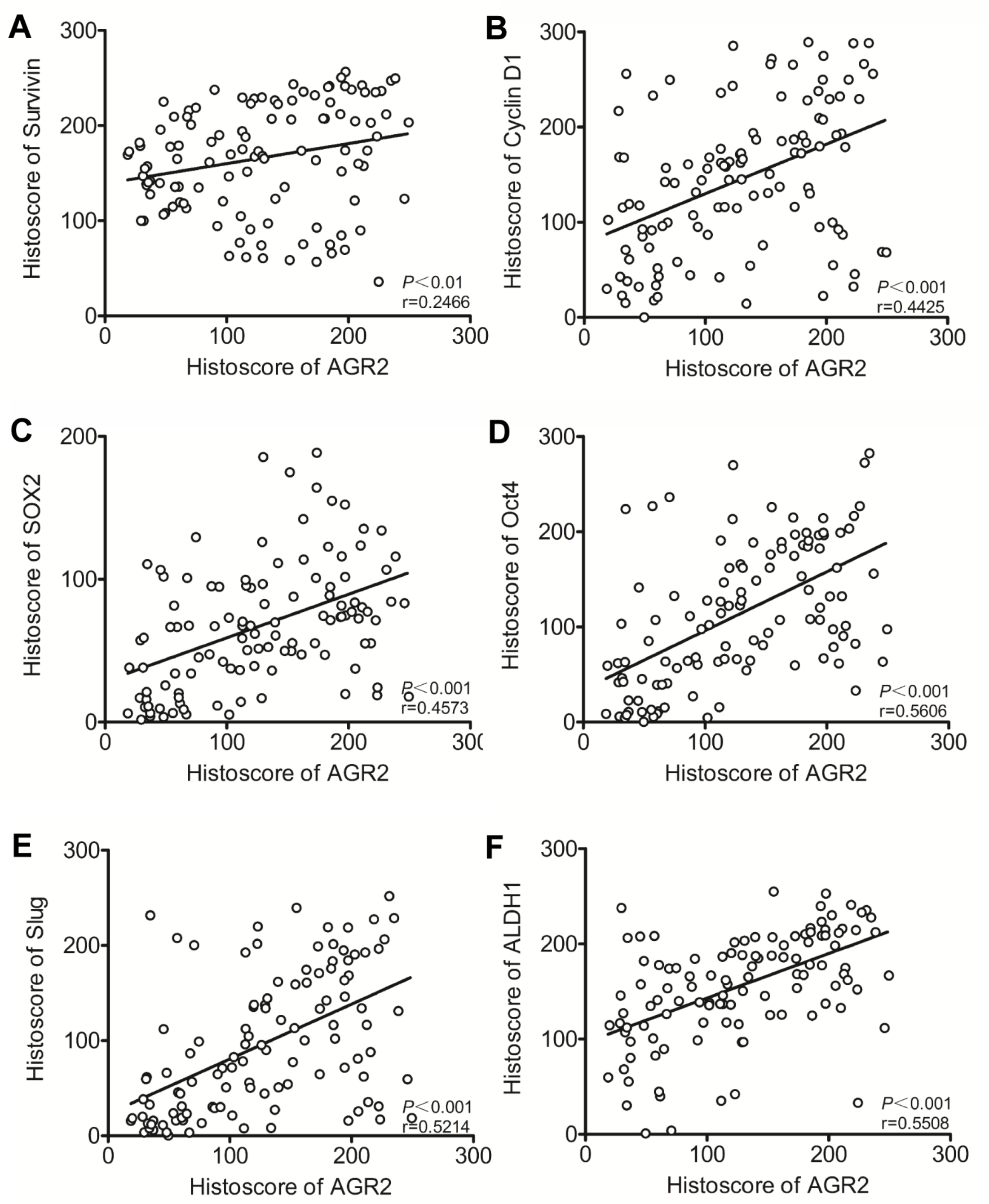

Supplementary Figure S3: Quantification and correlation of AGR2 with Survivin, Cyclin D1, ALDH1, Oct4, Sox2 and Slug in human HNSCC tissue. Correlation of AGR2 with Survivin (A), Cyclin D1 (B), Sox2 (C), Oct4 (D), Slug (E) and ALDH1 (F) in human HNSCC tissue array.

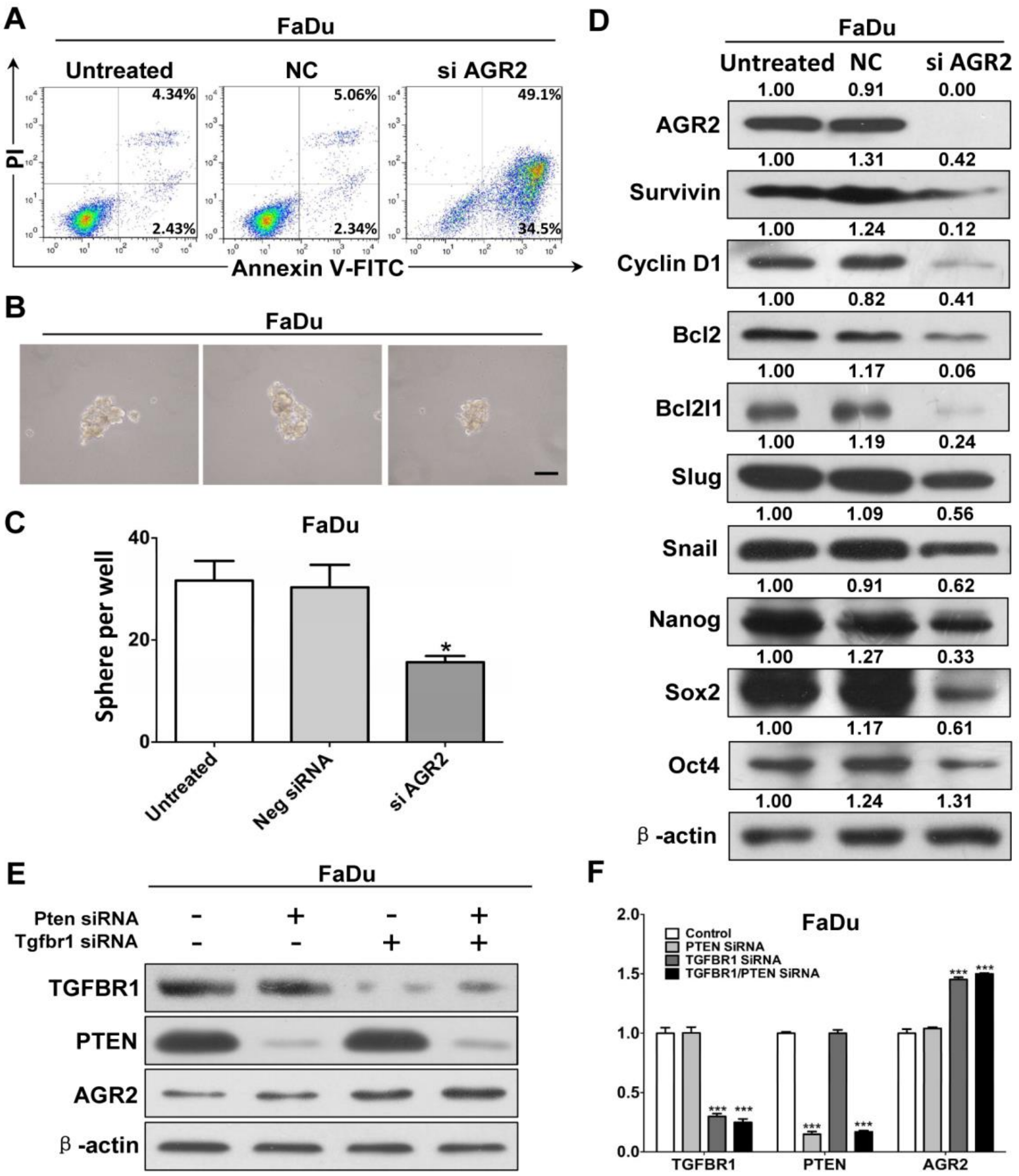

Supplementary Figure S4: Knock down of *AGR2* induces cell apoptosis and reduces colony formation in FaDu cell line. (A) Annexin V-FITC/PI dual labeling assay showed *AGR2* siRNA enhance apoptosis in FaDu cell lines by using flow cytometry; (B) Knock down of *AGR2* in FaDu cell line reduced sphere formation with quantification in (C); Scale bar=100μm;(D) Western blot analysis revealed that the protein level of Survivin, Cyclin D1, Bcl2, Bcl2l1, Slug, Snail, Nanog, Sox2 and OCT4 were reduced in different degrees after *AGR2* konck down in FaDu cell line for

48h.Quantification is performed using Image J by pixel analysis of band by normalized of β-actin as a loading control. Neg siRNA, negative siRNA,si *AGR2*, *AGR2* siRNA.(E) Western blot analysis of AGR2 48h after knocking down *PTEN*, *TGFBR1*, and combined *TGFBR1/PTEN* by using siRNA in FaDu cell line; (F) Quantitative analysis showed the protein level of *AGR2* in TGFBR1 siRNA group and *TGFBR1/PTEN* combined siRNA group were significantly higher than the control group ($P < 0.001$). Mean±SEM, ***, $P<0.001$.

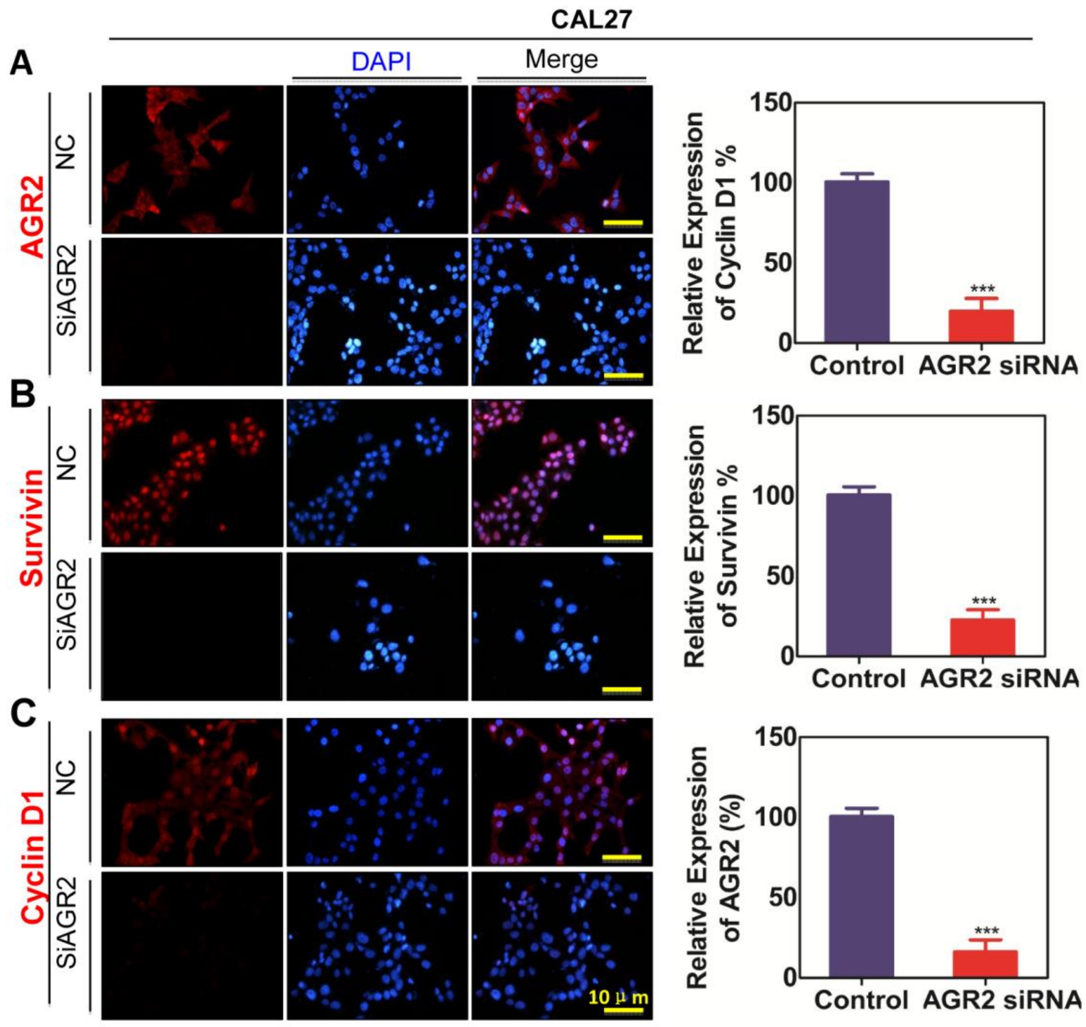

Supplementary Figure S5: Immunoflurosence of *AGR2* knock down reduce the expression of Survivin and Cyclin D1 in CAL27 cell line. AGR2 siRNA significantly reduce the expression of AGR2 (A), Survivin (B) and Cyclin D1 (C) in CAL27 cell line ($P < 0.001$, respectively). ***, $P < 0.001$.

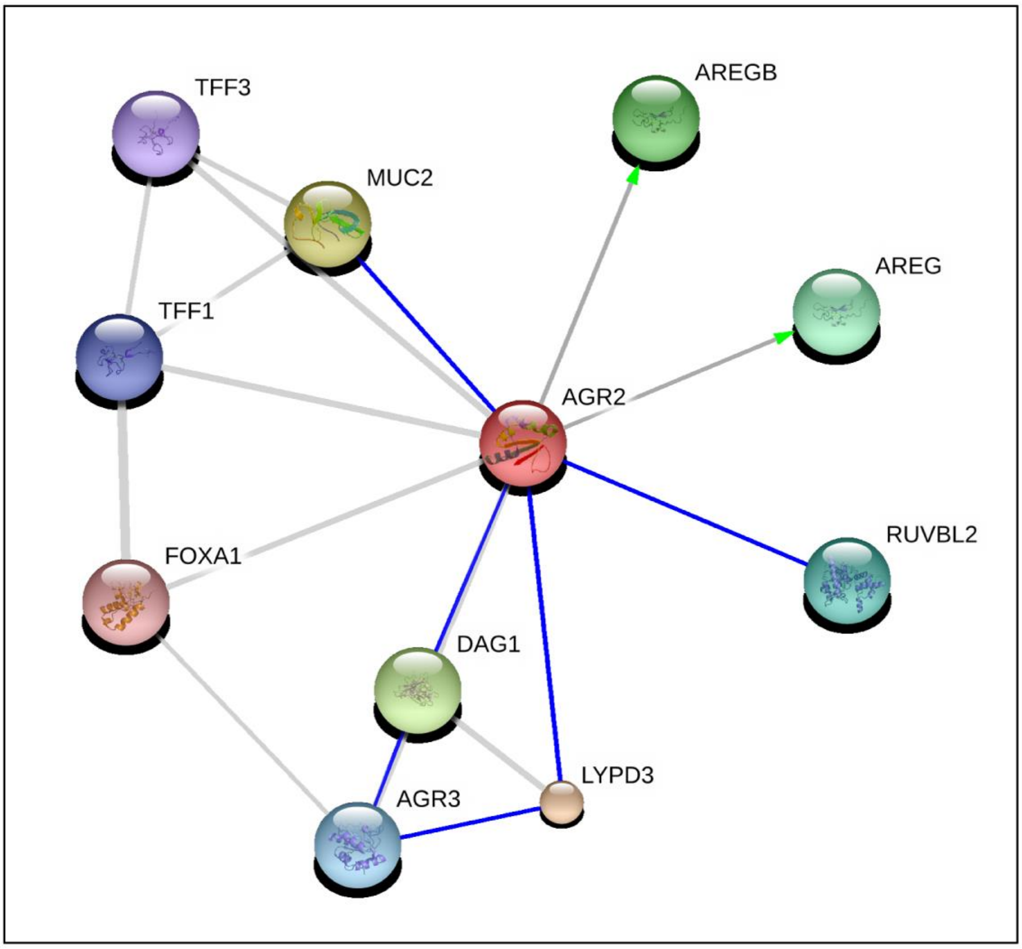

Supplementary Figure S6: Predict protein interaction of AGR2.

Computer predicts protein with direct protein-protein interaction with AGR2 analyzed by STRING database.

Supplementary Table 1: Cinicopathological features of 59 oral squamous cell carcinoma used in this study.

Case	*Location*	*Tumor(cm)*	*TNM*	*Grade*
1	Tongue	3.0*2.0	T2N0M0	I
2	Gingiva	2.0*1.5	T1N1M0	I
3	Tongue	1.5*1.5	T1N1M0	I
4	Tongue	2.0*2.0	T2N0M0	I
5	Gingiva	3.0*2.0	T2N0M0	I
6	Tongue	1.7*1.7	T1N0M0	I
7[†]	Tongue	3.0*1.5	T2N1M0	I
8	Tongue	2.0*2.0	T1N0M0	I
9	Gingiva	3.0*2.0	T2N0M0	I
10	Buccal mucosa	3.0*2.0	T2N1M0	I
11	Buccal mucosa	3.0*3.0	T2N0M0	II
12	Buccal mucosa	4.0*3.0	T3N1M0	II
13	Buccal mucosa	5.0*4.0	T3N0M0	II
14	Tongue	3.0*2.0	T2N1M0	II
15	Tongue	4.0*3.0	T3N0M0	II
16	Tongue	5.0*4.0	T3N0M0	II
17	Tongue	3.0*2.0	T2N0M0	II
18	Tongue	3.0*2.0	T2N1M0	II
19	Tongue	1.5*1.0	T1N1M0	II
20	Tongue	3.0*2.0	T2N1M0	II
21[†]	Tongue	3.0*2.0	T2N1M0	II
22	Tongue	3.0*2.5	T2N0M0	II
23	Tongue	4.0*2.0	T3N0M0	II
24	Tongue	3.0*2.0	T2N1M0	II
25	Oropharyngeal	3.0*3.0	T2N0M0	II
26	Mouth floor	2.0*2.0	T2N0M0	II
27	Gingiva	3.0*1.5	T2N0M0	III
28	Buccal mucosa	4.0*3.0	T3N1M0	III
29	Tongue	2.0*1.0	T2N0M0	III
30	Buccal mucosa	3.0*2.5	T2N1M0	III
31	Gingiva	3.0*2.5	T2N0M0	III
32	Gingiva	3.0*2.0	T2N0M0	III
33[†]	Tongue	5.0*3.0	T3N1M0	III
34	Mouth floor	3.0*3.0	T2N1M0	III
35[†]	Tongue	5.0*3.0	T3N1M0	III
36	Tongue	5.0*4.0	T3N0M0	III
37	Mouth floor	4.0*4.0	T3N0M0	III
38	Tongue	2.5*1.5	T2N0M0	I
39	Gingiva	3.0*1.5	T2N0M0	I

40	Tongue	1.5*1.5	T1N0M0	I
41	Mouth floor	3.0*2.5	T2N0M0	I
42	Gingiva	6.0*3.0	T3N0M0	I
43	Gingiva	2.0*2.0	T2N1M0	I
44	Gingiva	2.5*2.0	T2N0M0	II
45	Palate	3.0*2.5	T2N0M0	II
46	Mouth floor	2.0*2.0	T1N0M0	II
47[†]	Mouth floor	2.5*1.5	T2N1M0	II
48	Oropharyngeal	4.0*2.0	T2N0M0	II
49	Oropharyngeal	4.0*3.0	T2N0M0	II
50	Gingiva	3.0*3.0	T2N0M0	II
51	Tongue	2.5*1.0	T2N1M0	III
52	Mouth floor	4.0*3.0	T2N0M0	III
53	Oropharyngeal	4.0*3.0	T2N0M0	III
54	Palate	6.0*3.0	T3N0M0	III
55	Palate	6.0*3.0	T3N0M0	III
56	Gingiva	4.6*3.2	T2N0M0	III
57	Tongue	2.0*2.0	T1N0M0	III
58	Buccal mucosa	3.0*3.0	T2N1M0	III
59	Tongue	1.0*1.0	T1N0M0	III
60[*]	Oropharyngeal	2.5*2.0		
61[*]	Mouth floor	1.5*1.0		
62[*]	Oropharyngeal	3.0*2.0		
63[*]	Gingiva	4.0*4.0		
64[*]	Mouth floor	5.0*3.0		
65[#]	Lip	2.5*2.0		
66[#]	Buccal mucosa	3.0*2.5		
67[#]	Buccal mucosa	3.7*3.2		
68[#]	Gingiva	4.0*4.0		
69[#]	Tongue	5.0*1.0		
70[#]	Gingiva	4.0*4.0		
71[#]	Buccal mucosa	5.0*5.0		
72[#]	Tongue	2.0*2.0		
73[#]	Tongue	1.5*1.5		
74[#]	Buccal mucosa	4.0*3.0		
75[#]	Tongue	2.0*2.0		
76[#]	Gingiva	3.0*3.0		

[†]Specimen including original tumor as well as metastatic lymph nodes

[*] Recurrence after radiotherapy or radiotherapy before surgery

[#] Specimen including biopsy as well as surgical removal specimen after TPF chemotherapy

Supplementary Table 2: Pearson correlation coefficient test analyses of the array immunostainings of AGR2, Survivin, Cyclin D1, ALDH1, Sox2, Oct4 and Slug in OSCC (n=111).

Markers	AGR2	Survivin	Cyclin D1	ALDH1	Sox2	Oct4	Slug
AGR2		P<0.001 r=0.246	P<0.001 r=0.442	P<0.001 r=0.521	P<0.001 r=0.551	P<0.001 r=0.561	P<0.001 r=0.457
Survivin			P<0.001 r=0.452	P<0.001 r=0.460	P<0.001 r=0.459	P<0.001 r=0.478	
Cyclin D1				P<0.001 r=0.846	P<0.001 r=0.529	P<0.001 r=0.829	P<0.001 r=0.417
ALDH1					P<0.001 r=0.574	P<0.001 r=0.931	P<0.001 r=0.536
Sox2						P<0.001 r=0.579	P<0.001 r=0.379
Oct4							P<0.001 r=0.531
